# Novel pH sensing semiconductor for point-of-care detection of HIV-1 viremia

**DOI:** 10.1038/srep36000

**Published:** 2016-11-10

**Authors:** R. Gurrala, Z. Lang, L. Shepherd, D. Davidson, E. Harrison, M. McClure, S. Kaye, C. Toumazou, G. S. Cooke

**Affiliations:** 1 Division of Infectious Diseases, Imperial College, London, England; 2 DNA Electronics Ltd, Wood Lane, London, England; 3 Department of Bioengineering, Imperial College, London, England

## Abstract

The timely detection of viremia in HIV-infected patients receiving antiviral treatment is key to ensuring effective therapy and preventing the emergence of drug resistance. In high HIV burden settings, the cost and complexity of diagnostics limit their availability. We have developed a novel complementary metal-oxide semiconductor (CMOS) chip based, pH-mediated, point-of-care HIV-1 viral load monitoring assay that simultaneously amplifies and detects HIV-1 RNA. A novel low-buffer HIV-1 pH-LAMP (loop-mediated isothermal amplification) assay was optimised and incorporated into a pH sensitive CMOS chip. Screening of 991 clinical samples (164 on the chip) yielded a sensitivity of 95% (*in vitro*) and 88.8% (on-chip) at >1000 RNA copies/reaction across a broad spectrum of HIV-1 viral clades. Median time to detection was 20.8 minutes in samples with >1000 copies RNA. The sensitivity, specificity and reproducibility are close to that required to produce a point-of-care device which would be of benefit in resource poor regions, and could be performed on an USB stick or similar low power device.

Most of the 39 million individuals infected with HIV-1 live in sub-Saharan Africa in countries with limited infrastructure for healthcare delivery^1^,^2^. Whilst the key to reducing HIV-1 mortality is access to combination antiretroviral therapy (cART), regular monitoring is required to ensure effective therapy and detect treatment failure as early as possible in order to minimize the emergence of drug resistant virus^3^.

In well-resourced settings, the cornerstone of monitoring requires regular HIV-1 viral load assays (viral RNA copies per ml of plasma), usually performed on a large laboratory platform device^4^. Access to such technology is limited in many countries with a high HIV-1 burden and, where available, patients may have to make challenging return journeys to their clinics to receive results. Alternatives to viral load testing, such as measuring CD4 count, have limitations^5^. The challenges that inaccessible, and often unaffordable, viral load technologies pose to the delivery of high quality care for HIV-1 patients are now well recognised and improving diagnostics is now a key part of global strategies to combat the infection^6^.

The pipeline for novel viral load detection technologies is becoming more dynamic. Several devices are now available that are close to market^1^,^7^,^8^,^9^, others are in development. Despite this, most of these devices require a desktop instrument and laboratory processing which, whilst it has the potential to reduce costs, is still likely to limit access.

Here we describe a novel approach to HIV-1 viral load detection based on a novel CMOS chip platform that is able to detect small changes in pH generated by on-chip reactions^10^. The chip has embedded heaters and thermal sensors that facilitate on-chip nucleic acid amplification, and the hydrogen ions produced during DNA polymerisation are detected and quantified via the on board ISFET (Ion Sensing Field Effect Transistors) pH detection sensors (Fig. 1). The method is capable of working on a USB compatible stick without the need for additional power supply, labelling or fluorescence detection and with the potential for operation without a desktop platform. We describe the design and evaluation of an isothermal, low buffer, pH RT-LAMP reaction and demonstration of its efficacy with the CMOS technology.

## Results

### Optimisation of the pH RT-LAMP reaction conditions

To optimise the reaction conditions, cloned RNA standards were amplified in pH RT-LAMP tube reactions with varying concentrations of deoxynucleotides (dNTPs), betaine, MgSO_4_, and KCl. (Supplementary Fig. 1a–d). All reactions were carried out in triplicate with an input of 1000 RNA copies at 60 °C for 30 minutes. From these experiments, concentrations of 1 mM dNTPs, 0.4 mM betaine, 6 mM MgSO_4_ and 50 mM KCl were selected for use in subsequent pH RT-LAMP assays.

### Validation of pH RT-LAMP assay on RNA standards

**Figure 2:**
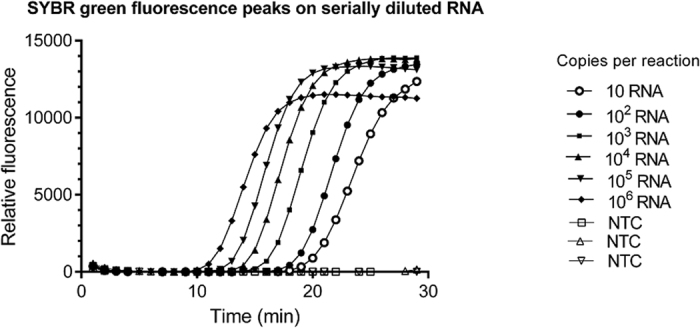
Real-time output of pH RT-LAMP reactions with varying input of cloned RNA target.

Sensitivity of the optimised pH RT-LAMP assay was determined by 10-fold serial dilution of cloned RNA. Results are shown in Fig. 2. The lowest detection limit of 10 RNA copies were successfully verified in a series of quadruplicate RT-LAMP reactions performed on 3 separate occasions. These experiments demonstrated that the pH RT-LAMP reaction in a tube assay could detect 10 RNA copies within the stipulated time. The observation of occasional low-level signals from negative reactions defined the cut-off time for a positive reaction as <30 minutes.

### Validation of pH RT-LAMP assay on HIV-1 RNA extracted from human plasma

**Figure 3:**
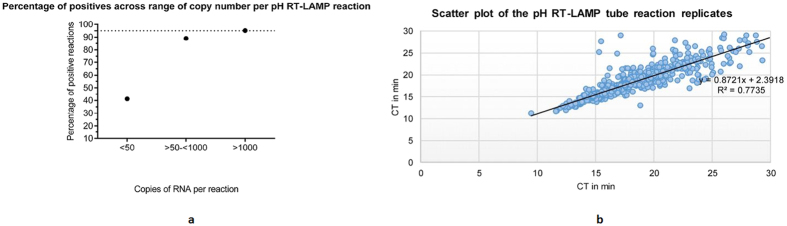
(**a**) Percentage positive reactions detected by pH RT-LAMP tube assays on HIV-1 RNA, extracted from human plasma. The dotted line is indicative of the percentage of positive reactions in plasma samples containing >1000 RNA copies/ml. (**b)** Shows the intra assay variability of duplicate RT-LAMP tube reactions on clinical samples.

HIV-1 RNA extracts from 991 plasma samples covering a wide range of clades and copy numbers were tested in the pH RT-LAMP tube assay in duplicate. Figure 3a shows the percentages of positive reactions plotted against template RNA copies per reaction. The detection rate for inputs >1000 copies/reaction was 95%, for inputs of 50 to 1000 copies, 88.7% and for inputs <50 copies/reaction 41.2%. Figure 3b shows the intra-assay variability for the duplicate samples.

### pH change in RT-LAMP assay

**Figure 4:**
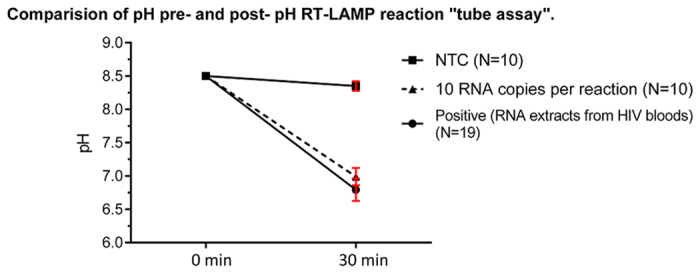
Shows the drop in the pH between negative and positive pH RT-LAMP reactions. Dotted line represents reactions performed with cloned RNA and normal line represents those performed with RNA extracted from HIV-1 infected bloods. Screen shots of the chip Graphic user interface (GUI).

The drop in the pH post-amplification observed in the positive reactions was measured in RT-LAMP assays on RNA standards and 19 RNA extracts from human plasma. Figure 4 shows the pH pre- and post-amplification, for positive reactions and no-template reaction controls [NTC] (water).

### Validation of chip pH RT-LAMP assays on RNA from human plasma

**Figure 5:**
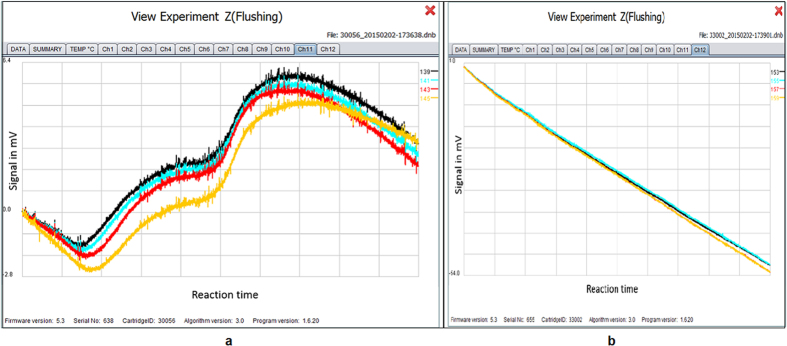
Chip output data from positive **(a)** and negative **(b)** samples. The positive inflection seen in positive samples is a consequence of detection of falling pH.

HIV-1 RNA extracted from 164 clinical plasma samples was analysed in the on-chip pH LAMP assay together with 18 NTCs. All reactions were performed at 60 °C for 50 minutes. A typical signal output from the detection chip is shown in Fig. 5. The proportions of positive reactions were 31/35 (88.8%) for inputs >1000 copies, 48/63 (76.1%) 50–1000 copies and 14/66 (21.2%) <50 copies. Data are shown in Table 1.

## Discussion

We describe proof-of concept for a novel method for point-of-care (POC) detection of HIV-1 viremia. We show that RT pH-LAMP is feasible in low buffer conditions, performs well across a wide range of clinical isolates and, through electronic detection of pH change, is able to distinguish positive from negative samples when run on a CMOS chip. The system as evaluated incorporates many of the requirements for an assay that can be used in field conditions with minimal laboratory infrastructure, including the lack of a need for refrigeration. The USB mounted device can potentially operate with any suitably configured hand-held computer device and has no other requirement for a mains power supply. In addition, the system needs no complex equipment requiring skilled calibration or maintenance and utilises a self-contained, disposable detection system which is potentially very cheap to manufacture by standard silicon-chip production methods. The underlying approach has been applied to Ion Torrent DNA sequencing technology^11^ and underpins a commercial DNA testing service^12^. However, this is the first report of pH mediated on-chip detection of RNA.

The development of HIV RT pH-LAMP was chosen over other amplification methods for three reasons; the isothermal nature of the reaction simplifies the technical challenges of thermal cycling on the chip (though such cycling is possible) and, more importantly, the use of LAMP generates higher concentrations of amplification products^13^ which, through greater changes in pH, improves the sensitivity and speed of the assay. Thirdly, LAMP is less perturbed by non-specific inhibitors than PCR^14^, allowing the potential for less stringent RNA preparation in a final integrated device, unlike PCR. LAMP has previously been used to detect HIV-1 RNA using alternative read-out platforms, such as turbidimetry and fluorimetry^15^. One advantage of the approach described here is the potential to multiplex different assays on one CMOS chip. After evaluating targets (and combinations of targets) in the HIV-1 *gag* and *pol* genes, our method uses primer sequences targeted to the integrase gene in common with published LAMP methods and laboratory-based commercial PCR assays. In addition, we were able to meet the challenges or viral diversity and improve sensitivity across a wide range of clinical isolates through the use of redundant bases and inosine.

The data generated show that the RT pH-LAMP reaction, as designed, is capable of detecting low copy numbers (down to 10 RNA copies/reaction) of RNA transcripts cloned from the HIV-1 integrase gene. The time to generate a positive reaction was 30 minutes using fluorescent detection, extending to a maximum of 50 minutes when the reaction was performed on the pH-sensitive silicon chip with low copy number. The cases of false negative results at this detection limit were not biased to any particular HIV-1 clade (Supplementary Table 3a,b) and sequencing studies (not published) could not determine any LAMP primer mismatches that could explain the failure to amplify.

While laboratory based HIV-1 viral load detection systems in well-resourced clinical settings are able to detect an HIV-1 viral load of 50 copies/ml or lower, such sensitivity is not required for the monitoring of ART in resource-poor settings. Modelling demonstrates that thresholds of 1000 copies/ml or even 5000 copies/ml may be acceptable^16^ and more cost-effective and WHO recommendations suggest that detection of viral loads >1000 copies/ml have sufficient sensitivity^17^. The detection rate of 95% for the LAMP reaction at >1000 copies/reaction using fluorescent detection is encouraging, but before the method can be considered suitable for field use, it will need to be integrated with an efficient RNA extraction method capable of achieving high yields of nucleic acid from peripheral blood. The optimisation data here shows that high sensitivity can be achieved if sufficient target RNA is in the assay. The sensitivity of the assay on the chip was slightly lower than that seen *in vitro* (88.8% and 95% respectively) (Table 1) which is most likely a consequence of the low reaction volume (12 × 0.6 μl/reaction) compared to the fluorescent output (1 × 25 μl) and this may account for some reduction in the rate of detection. As well as maximising the quantity of viral nucleic acid that can be delivered to the chip, further optimisation of the size of the reaction chambers is planned which could potentially improve sensitivity further. A second generation chip that can hold more than 7.2 μl/reaction is being tested for deployment and it is anticipated that it would match the performance of the fluorescence based RT-LAMP for HIV-1.

Whilst further development is required, including an evaluation of specificity across a wide range of clinical isolates before field evaluations, our HIV specific pH-LAMP assay coupled with novel CMOS chip technology shows great potential as a route to a point of care diagnostic suitable for use in clinical settings without access to a laboratory infrastructure, as is often the case in the developing world. The technology has the potential to be scalable for the detection of multiple pathogens simultaneously and this will be part of future programme of work.

## Methods

### Synthesis of RNA standards

Preliminary RT-LAMP assay development and optimisation was undertaken using cloned RNA transcripts of the complete HIV-1 integrase (IN) coding region. The IN coding region of the 8E5 strain of HIV-1 (clade B) was amplified by nested PCR using primers 5′-CTCACAGTATGCATTAGGAATYAT and 5′-CCTTATGGCAGATTCTGAAAAACA in the first round and 5′-GCACACAAAGGAATTGGAGGAAAT and 5′-TAGTGGGATGTGTACTTCTGAACT in the second round. The PCR products were cloned into the pCR 2.1-TOPO Vector (Life Technologies, Paisley, UK). DNA was extracted using a Plasmid Minikit (Qiagen, Manchester, UK) and the plasmid linearized by digestion with restriction enzyme Pst1 (New England Biolabs, Hitchin, UK). RNA transcripts were made using the MEGAScript T7 polymerase kit (Life Technologies) according to the manufacturer’s instructions. Post-transcription, the nucleic acid was DNAse digested (DNAse 1 amplification grade, Life Technologies) for 30 minutes at 37 °C and RNA extracted with the QIAamp viral RNA kit (Qiagen).

### pH RT-LAMP

The pH RT-LAMP assay was initially developed and optimised on a real-time thermal cycler by including SYBR- Green dye in the reaction mix (referred to as the “tube assay”). Validation of the developed method was performed using both cloned RNA and RNA extracts from clinical samples on the real-time thermal cycler and the CMOS chip platform. To allow detection on the chip platform, a modified master-mix formulation that minimises buffering (pH-LAMP) was used, and the reactions included reverse transcriptase to allow amplification of the RNA target (pH RT-LAMP).

Primer sequences used throughout are given in Supplementary Table 1. The final optimised reaction master-mix contained 50 mM KCl, 2.5 mM NH_4_Cl, 3% Siloxane (Invitrogen, USA), 1 mg/ml BSA, 0.4 M betaine (Sigma-Aldrich, Dorset, UK), 1 mM dNTPs, 6 mM MgSO_4_, 0.2 mM NaOH, 0.4x SYBR Green [stock 10,000x] (Life Technologies), 0.2 μM of each outer primer, F3 and R3, 1.6 μM of each inner primer, FIP and RIP, 0.8 μM of each loop primer, FLP and RPL, 24U BST polymerase (New England Biolabs), 2.25U AMV reverse transcriptase (Life Technologies) and 10 μl template, with the final reaction volume adjusted to 25 μl with H_2_O. The pH RT-LAMP reaction conditions comprised of a one-step reverse transcription and DNA amplification at 60 °C for 30 minutes, with a reading taken every minute. The assay was performed on a real-time thermal cycler (MX3000*p*, Stratagene, USA). The pH changes in tube reactions were measured with a Titan handheld micro pH probe (Sentron, Leek, Netherlands) before and after the reaction took place. The master-mix for chip pH RT-LAMP assay was identical to the tube reactions excluding SYBR-green. Five times the volume (125 μl) of master-mix plus target was applied to the chip and the reaction was allowed to process for 50 minutes at 60 °C.

### PCR analysis of LAMP amplicons

LAMP reactions generate a distinctive ladder pattern when analysed by agarose gel electrophoresis. To verify the specificity of the pH RT-LAMP assay, the amplified products were diluted 10-fold in water and run on a 2% agarose gel stained with SYBR-Safe dye (Life Technologies). The specificity of LAMP amplicons was verified by PCR amplification. Briefly, 2 μl of the diluted RT- LAMP products were added to 10 μl 2x Fast Cycling PCR master-mix (Qiagen) containing 1 μl each of the forward primer, 5′-CAATTTTAAAAGAAAAGGGGGGATT and the reverse primer, 5′-TACTGCCCCTTCACCTTTCCA in a final 20 μl reaction volume. The PCR reactions were carried out with an initial denaturation step of 95 °C for 15 minutes followed by 40 cycles of 94 °C for 10 seconds and 60 °C for 60 seconds. PCR products were visualised by agarose gel electrophoresis.

### Clinical samples

To validate the tube assay, HIV-1 RNA was extracted from 991 human plasma samples with detectable viral load assayed by a commercial assay (Versant v3.0, Bayer). The use of anonymised residual samples from routine HIV viral load testing for assay development was approved by the local research ethics committee of St Mary’s Hospital Paddington and the study used materials provided by the local Communicable Disease Research Tissue Bank (Ethics refs H15/SC/0089, 09/H0606/106). RNA from human plasma was extracted using the Viral RNA Minikit (Qiagen) in accordance with approved guidelines. The clade distribution of the samples, derived from protease-RT sequence data generated in routine resistance testing, is shown in Supplementary Table 2. For validation of the assay on the CMOS chip, RNA from 164 samples was extracted.

### pH RT-LAMP on CMOS chip

The CMOS chip used consists of 4 smaller quad chips, each containing 3 micro fluidic chambers that individually hold a reaction volume of 0.6 μl, giving a total reaction volume on the chip of 7.2 μl. The RT-LAMP mixture was pipetted through the opening at the top and was uniformly distributed to all chambers via the micro fluidic channels. The chip was inserted into the chip holder and reaction was powered via a standard USB port on a PC. Bespoke software on the PC converted the pH data received by the ISFETs to filtered digital information, which could be visualised real-time on the screen via graphic user interface in the form of a plot of the mV of signal produced and the time of reaction in seconds. A chip reaction is considered positive if ≥1 chambers produces pH inflection within 50 minutes of run time.

## Additional Information

**How to cite this article**: Gurrala, R. *et al*. Novel pH sensing semiconductor for point-of-care detection of HIV-1 viremia. *Sci. Rep.* **6**, 36000; doi: 10.1038/srep36000 (2016).

**Publisher’s note**: Springer Nature remains neutral with regard to jurisdictional claims in published maps and institutional affiliations.

## Supplementary Material

Supplementary Information

## Figures and Tables

**Figure 1:**
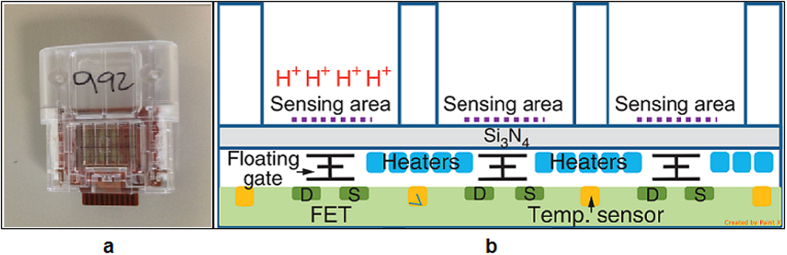
**(a)** Image of prototype chip for amplification and detection of nucleic acids compatible with a USB port. **(b)** Schematic of a chip. Each chamber functions independently, when the pH of the chamber changes the ISFET (ion sensitive field effect transistor) generates an electrical signal.

**Table 1:** Tabulated results of the tube and chip RT-LAMP reactions on clinical samples.

pH RT-LAMP “tube assay”	Copies per reaction	<50	>50–<1000	>1000
	Total samples per category	182	320	489
	Number of positives	75	284	465
	**% sensitivity**	**41.20%**	**88.75%**	**95.00%**
NTC	Positive reactions/total	0/18		
	**% specificity**	**100%**		
				
pH RT-LAMP “on-chip assay”	Copies per reaction	<50	>50–<1000	>1000
	Total samples per category	66	63	35
	Number of positives	14	48	31
	**% sensitivity**	**21.20%**	**76.10%**	**88.80%**
	Median time (in minutes) to the first positive chamber	**32.3**	**27.09**	**20.8**
	Median signal in mV	**9.25**	**9.62**	**10.93**
	% of chambers amplified	**27.22%**	**37.13%**	**63.80%**
NTC	Positive reactions/total	0/18		
HIV undetectable plasma	Positive reactions/total	0/13		
	**% specificity**	**100%**		

## References

[b1] UNAIDS, Global AIDS Update. http://www.unaids.org/sites/default/files/media_asset/global-AIDS-update-2016_en.pdf (2016) (accessed on 04 July 2016).

[b2] WHO, HIV/AIDS. http://www.who.int/mediacentre/factsheets/fs360/en/ (2016) (accessed on 04 July 2016).

[b3] Phillips A. . Sustainable HIV treatment in Africa through viral-load-informed differentiated care. Nature 528, S68–S76 (2015). 26633768 10.1038/nature16046 PMC4932825

[b4] UNITAID, Implementation of CD4 and viral load testing in decentralized, remote and resource-limited settings in MSF HIV programmes. http://www.unitaid.eu/en/cd4 (2014) (accessed on 04 July 2016).

[b5] Bisson G. P. . Diagnostic accuracy of CD4 cell count increase for virologic response after initiating highly active antiretroviral therapy. AIDS 20, 1613–1619 (2006). 16868442 10.1097/01.aids.0000238407.00874.dc

[b6] UNAIDS, The Treatment 2.0 Framework for Action: Catalysing the Next Phase of Treatment, Care and Support. http://apps.who.int/iris/bitstream/10665/44640/1/9789241501934_eng.pdf (2011) (accessed on 04 july, 2016).

[b7] Tanriverdi S. . A rapid and automated sample-to-result HIV load test for near-patient application. J Infect Dis 201, S52–S58 (2010). 20225947 10.1086/650387

[b8] Ritchie A. V. . SAMBA HIV semiquantitative test, a new point-of-care viral-load-monitoring assay for resource-limited settings. J Clin Microbiol 52 **(9)**, 3377–3383 (2014). 25031444 10.1128/JCM.00593-14 PMC4313165

[b9] Jani I. V. . Accurate early infant HIV diagnosis in primary health clinics using a point-of-care nucleic acid test. J Acquir Immune Defic Syndrome 67, e1–e4 (2014). 10.1097/QAI.0000000000000250 24933096

[b10] Toumazou C. . Simultaneous DNA amplification and detection using a pH-sensing semiconductor system. Nat Methods 10 **(7)**, 641–646 (2013). 23749303 10.1038/nmeth.2520

[b11] Jonathan M. . An integrated semiconductor device enabling non-optical genome sequencing. Nature 475, 348–352 (2011). 21776081 10.1038/nature10242

[b12] Denovan N. DNA Electronics Partners With Geneonyx To Offer GenalysisÂ^®^ Real-Time DNA Detection Technology For Genetics-Driven Retail Services. Reuters http://www.reuters.com/article/idUS120862+19-Mar-2012+BW20120319 (2012) (accessed on 04 July 2016).

[b13] Notomi T. . Loop-mediated isothermal amplification of DNA. Nucleic Acids Res 15 **(28)**, 12 (2000). 10.1093/nar/28.12.e63 PMC102748 10871386

[b14] Villari C. . Use of loop-mediated isothermal amplification for detection of Ophiostoma clavatum, the primary blue stain fungus associated with Ips acuminatus. Appl. Environ. Microbiol 79 **(8)**, 2527–2533 (2013). 23396326 10.1128/AEM.03612-12 PMC3623199

[b15] Rudolph D. L. . Detection of acute HIV-1 Infection by RT-LAMP. PLoS One 10 **(5)** (2015). 10.1371/journal.pone.0126609 PMC4439053 25993381

[b16] Fox M. P. . Rates and predictors of failure of first-line antiretroviral therapy and switch to second-line ART in South Africa. J Acquir Immune Defic Syndrome 60 **(4)**, 428–437 (2012). 10.1097/QAI.0b013e3182557785 PMC3392418 22433846

[b17] Bennett D. E. . The World Health Organization’s global strategy for prevention and assessment of HIV drug resistance. Antivir Ther 13 **(2)**, 1–13 (2008). 18578063

