# Supplementary material for: Novel pH sensing semiconductor for point-of-care detection of HIV-1 viremia
Source: Sci Rep. 2016 Nov 10;6:36000. doi: 10.1038/srep36000 (PMC5103182; doi:10.1038/srep36000)

**Supplementary material for**

# Novel pH sensing semiconductor for point-of-care detection of HIV-1 viremia

Gurrala, R[1], Lang, Z[2], Shepherd, L[2], Davidson, D[2], Harrison, E[2], McClure, M[1], Kaye, S[1], Toumazou, C[2,3], Cooke, GS[1]

1 Division of Infectious Diseases, Imperial College, London
2 DNA Electronics Ltd, Wood Lane, London
3 Department of Bioengineering, Imperial College, London

# Supplementary data

**Supplementary table 1-** Primer sequences used in pH-LAMP reactions generating a primary amplicon of 317bp

| Primer designation | Sequence | Location[1] |
|---|---|---|
| F3 | 5'- CCC TAC AAT CCC CAA AG – 3' | 4199-4215 |
| F2 | 5'- ATG GCA GTA TTC ATY CAC AAT TTT AA– 3' | 4307-4332 |
| FLP | 5'- CCA ATC CCC CCT TTT– 3' | 4336-4350 |
| F1C | 5'- TAT YCT I[2]TC CCC TGC ACT GTA CCC C– 3' | 4351-4370 |
| R1C | 5'- CGG GTT TAT TAC A– 3' | 4445-4457 |
| RLP | 5'- GAC AGC AGA GAI CC– 3' | 4460-4473 |
| R2 | 5'- AGY TTT GCT GGT CCT TTC CAA– 3' | 4477-4497 |
| R3 | 5'- CCT TCA CCT TTC CA– 3' | 4502-4515 |
| FIP | 5'-TAT YCT 6TC CCC TGC ACT GTA CCC CTT TT[3]A TGG CAG TAT TCA TYC ACA ATT TTA A – 3' | |
| RIP | 5'-CGG GTT TAT TAC ATT TTA GYT TTG CTG GTC CTT TCC AA– 3' | |

[1] Location in GenBank K03455 [2] inosine [3] thymidine spacer

**Supplementary table 2-** Clade distribution of clinical samples used for validation

| Clade | Samples analysed in “tube assay” | Samples analysed in “chip assay” |
|---|---|---|
| A | 52 (5.2%) | 14 (8.5%) |
| AE | 18 (1.8%) | 5 (3%) |
| AG | 58 (5.8%) | 16 (9.7%) |
| B | 581 (58.6%) | 19 (11.5%) |
| C | 135 (13.6%) | 83 (50.6%) |
| D | 34 (3.4%) | 8 (4.8%) |
| F | 6 (0.6%) | 0 |
| G | 21 (2.1%) | 4 (2.4%) |
| H | 2 (0.2%) | 0 |
| J | 4 (0.4%) | 0 |
| K | 1 (0.1%) | 0 |
| mixed | 79 (7.9%) | 10 (6%) |
| Unknown | 0 | 5 (3%) |
| Total | 991 | 164 |

**Supplementary table 3-** Comparison of clade distribution of samples included in the assays with failed pH RT-lamp tube assay reactions **(a)** and on-chip assay reactions **(b)** performed on clinical samples. Only the samples with viral load > 1000 copies per ml of plasma are represented above.

**a**

| Clade | Proportion of total | Proportion of failures |
|---|---|---|
| A | 5.2% | 0% |
| AG | 5.8% | 14.3% |
| B | 58.6% | 58.9% |
| C | 13.6% | 12.5% |
| D | 3.4% | 5.3% |
| Other | 13.6% | 8.9% |

**b**

| Clade | Proportion of total | Proportion of failures |
|---|---|---|
| A | 6.25% | 0% |
| AG | 10.4% | 10% |
| AE | 2.0% | 50% |
| B | 9.3% | 0% |
| C | 57.2% | 8.1% |
| D | 6.25% | 33.33% |
| G | 2.0% | 100% |
| Other | 6.25% | 16.6% |

**Supplementary figure 1a-d-** Optimisation of pH RT-LAMP reaction conditions for dNTP **(a)** Betaine **(b)**, $MgSO_4$ **(c)** and KCl **(d)**.  1000 copies of RNA standard was used as template for the reactions. The concentration that produced lowest time to detection in minutes was selected for the final protocol.

a

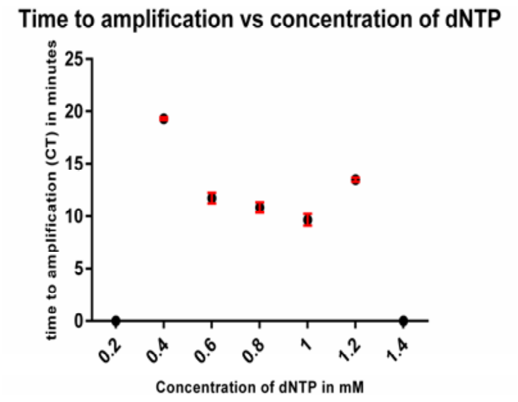


b

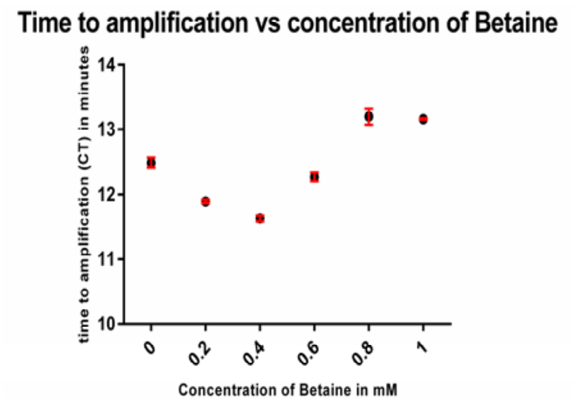


c

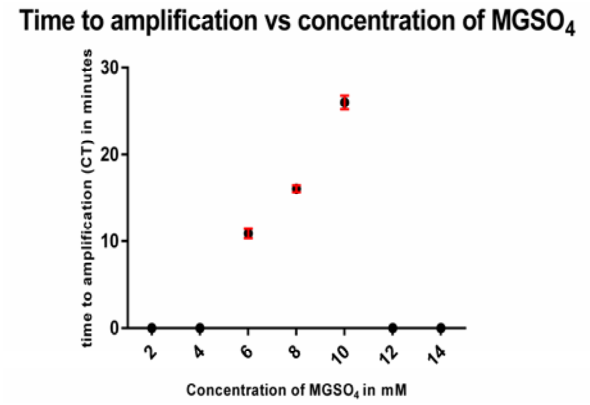


d

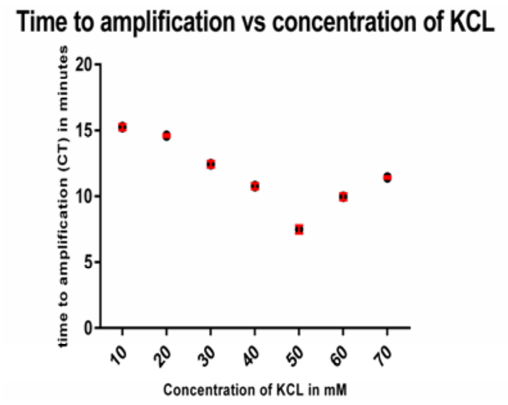

Supplement: Supplementary Information [file srep36000-s1.pdf]
